# Somatostatin and Neuropeptide Y in Cerebrospinal Fluid: Correlations With Amyloid Peptides Aβ_1–42_ and Tau Proteins in Elderly Patients With Mild Cognitive Impairment

**DOI:** 10.3389/fnagi.2018.00297

**Published:** 2018-10-01

**Authors:** Emmanuelle Duron, Jean-Sébastien Vidal, Dominique Grousselle, Audrey Gabelle, Sylvain Lehmann, Florence Pasquier, Stéphanie Bombois, Luc Buée, Bernadette Allinquant, Susanna Schraen-Maschke, Christiane Baret, Anne-Sophie Rigaud, Olivier Hanon, Jacques Epelbaum

**Affiliations:** ^1^AP-HP, Hôpital Broca, Service de Gériatrie, Paris, France; ^2^Université Sorbonne Paris Cité, UMR-S894, INSERM Université Paris Descartes, Centre de Psychiatrie et Neuroscience, Paris, France; ^3^APHP, Hôpital Paul Brousse, Service de Gériatrie du Dr Karoubi, Villejuif, France; ^4^Université Paris-Sud 11, Centre de Recherche en Épidemiologie et Santé des Population- Depression et Antidépresseurs, INSERM UMR-1178, Le Kremlin-Bicêtre, France; ^5^Université Paris Descartes, Sorbonne Paris Cité, Paris, France; ^6^Memory Research and Resources Center, Gui de Chauliac Hospital, University of Montpellier, Montpellier, France; ^7^Laboratoire de Biochimie Protéomique Clinique, CHU Montpellier, University of Montpellier, INSERM, Montpellier, France; ^8^University of Lille, INSERM 1171, CHU, Centre Mémoire (CMRR) Distalz, Lille, France; ^9^Univ Lille, Inserm, UMRS 1172, LabEx DISTALZ, Lille, France; ^10^UF de Neurobiologie, Centre Biologie Pathologie du CHU-Lille, Lille, France; ^11^MECADEV UMR 7179 CNRS, Muséum National d’Histoire Naturelle, Paris, France

**Keywords:** somatostatin, neuropeptide Y, peptides Aβ_1–42_, tau proteins, cerebrospinal fluid, mild cognitive impairment

## Abstract

A combination of low cerebrospinal fluid (CSF) Amyloid β_1–42_ (Aβ_1–42_) and high Total-Tau (T-Tau) and Phosphorylated-Tau (P-Tau) occurs at a prodromal stage of Alzheimer’s disease (AD) and recent findings suggest that network abnormalities and interneurons dysfunction contribute to cognitive deficits. Somatostatin (SOM) and Neuropeptide Y (NPY) are two neuropeptides which are expressed in GABAergic interneurons with different fates in AD the former only being markedly affected. The aim of this study was to analyze CSF SOM, NPY and CSF Aβ_1–42_; T-Tau, P-Tau relationships in 43 elderly mild cognitively impairment (MCI) participants from the Biomarker of AmyLoïd pepTide and AlZheimer’s disease Risk (BALTAZAR) cohort. In these samples, CSF SOM and CSF Aβ_1–42_ on the one hand, and CSF NPY and CSF T-Tau and P-Tau on the other hand are positively correlated. CSF SOM and NPY concentrations should be further investigated to determine if they can stand for early AD biomarkers.

Clinical Trial Registration: www.ClinicalTrials.gov, identifier #NCT01315639.

## Introduction

Alzheimer’s disease (AD) neuropathology is characterized by intraneuronal protein clusters of hyperphosphorylated Tau protein (neurofibrillary tangles) and extracellular amyloid beta (Aβ) protein aggregation that start decades before the occurrence of clinical symptoms (Bateman et al., 2012; Epelbaum et al., 2017). Research criteria for AD includes cerebrospinal fluid (CSF) pathophysiological markers (Dubois et al., 2014): low CSF Aβ_1–42_ concentration reflects the brain amyloid burden, high Total-Tau (T-Tau) CSF concentration reflects the intensity of neuronal loss and high Phosphorylated-Tau (P-Tau) is believed to be a direct marker of tangle pathology. The combination of low Aβ_1–42_ and high T-Tau or p-Tau, which occurs at a prodromal stage of AD (Hansson et al., 2006) and even before clinical impairment (Sutphen et al., 2015) has a sensitivity of 90%–95% and a specificity of about 90% in AD diagnosis (de Souza et al., 2011; Gabelle et al., 2013; Lehmann et al., 2014). In this way, an individual patient’s risk estimation of any-type dementia can be improved by adding CSF biomarker information to clinical and imaging tests as recommended in routine care (Handels et al., 2017; Albert et al., 2018).

Recent findings suggest that network abnormalities and interneuron dysfunction contribute to cognitive deficits (Palop and Mucke, 2016). Somatostatin (SOM) and Neuropeptide Y (NPY) are two neuropeptides, co-expressed in GABAergic interneurons in cortex and hippocampus and in brain areas implicated in learning and memory (Epelbaum et al., 2009; Borbély et al., 2013). SOM concentration and expression levels are decreased in the cortex and hippocampus of AD patients (Davies et al., 1980; Gahete et al., 2010) while NPY-positive neurons are reduced in AD hippocampus (Kowall and Beal, 1988). In experimental AD models, increasing expression of Aβ_1–42_ with aging is associated with an early fall of SOM interneurons in the hippocampus followed by NPY-positive ones (Saiz-Sanchez et al., 2016) or a reverse pattern (Wilcock et al., 2008; Albuquerque et al., 2015). Remarkably, SOM (in its 14 amino-acid moiety) was recently observed to be the most selectively enriched oligomeric Aβ_1–42_ binder (Wang et al., 2017). In addition, a low level of CSF SOM correlates with cognitive deficits in AD (Tamminga et al., 1987) while CSF NPY concentrations changes are not as well documented (Gabriel et al., 1993).

To date, the relationships between CSF Aβ_1–42_, T-Tau, P-Tau and CSF SOM and NPY concentrations have never been reported especially in subjects suffering from cognitive impairment. The aim of this study was to decipher the concentrations of SOM and NPY in elderly subjects with cognitive impairment and analyze these relationships with the other AD biomarkers.

## Materials and Methods

The Biomarker of AmyLoïd pepTide and AlZheimer’s disease Risk (BALTAZAR) study was a longitudinal, multicenter study including 23 French memory clinics. It was approved by the local ethics committee (ClinicalTrials.gov Identifier #NCT01315639). The BALTAZAR study aimed to study the relationships between plasmatic biomarkers (Aβ and soluble amyloid precursor protein α, sAPP_α_) and determine the relevance of these biomarkers on the risk of conversion of patient aged 70 years and older with mild cognitive impairment (MCI) according to international criteria (Petersen, 2004; Portet et al., 2006) towards AD stage. All study participants or their legal guardians provided written informed consents. The study protocol was approved by local ethics comity (Comité de Protection des Personnes, Ile de France IV Saint Louis Hospital) and conducted in accordance with the Declaration of Helsinki.

In a subsample ancillary study, we prospectively included consecutive subjects attending a geriatric day care hospital at the Broca hospital, suffering from MCI, who underwent a lumbar puncture, with CSF AD biomarkers measurements (Aβ_1–42_, T-Tau and P-Tau) and Magnetic Resonance Imaging (MRI) scans at baseline. Subjects with geriatric depression scale (GDS) score over 20/30 (a score over 15/30 indicating a risk of depression; Yesavage, 1988) were excluded.

### Clinical and Biological Data

Demographic data, vascular risks factors: hypertension defined as self-reported diagnosis of hypertension or use of antihypertensive medications or blood pressure >140/90 mmHg, diabetes defined as history of diabetes or use of glucose-modifying medications, cardio-vascular diseases (coronary heart disease, stroke, heart failure) and treatments were recorded by a physician. The educational level was scored as elementary school, secondary school and high school diploma and above. Each participant had a physical examination performed by a physician including the calculation of the body mass index (BMI).

Fasting blood samples were collected for all subjects (complete blood count, measurement of electrolytes, total cholesterol, thyroid stimulating hormone, folate and vitamin B12, fasting blood glucose, albumin, C-Reactive Protein (CRP). APOE was genotyped in a single centralized laboratory (Centre de Biologie-Pathologie, Lille University Hospital, France) using a classical polymerase chain reaction (PCR) digestion method.

CSF SOM and NPY concentrations were measured by radioimmunoassay as previously reported for SOM (Grouselle et al., 1998) and using a NPY radioimmunoassay kit (Phoenix, Strasbourg, France). CSF T-Tau, P-Tau and Aβ_1–42_ concentrations were measured in the same laboratory (team 33, Biochimie Hôpital St Eloi—IRB, Montpellier) using the commercial kits: INNOTEST^®^hTAU Ag, INNOTEST^®^β-Amyloid_(1–42)_ (Andreasen et al., 2001) and INNOTEST^®^Phospho-Tau_(181P)_ (Vanderstichele et al., 2006; Fujirebio^®^, Belgium). The quality of the results was ensured by using validated standard operating procedures (del Campo et al., 2012) and internal quality controls (QCs). The QC coefficients of variation obtained on the CSF analyses within each lot and between lots ranged consistently below 15%. In addition, external QC procedures were used to confirm the quality of our results.

### Cognitive Evaluation

All subjects underwent comprehensive cognitive assessment performed by trained neuropsychologists (Philippi et al., 2016). Episodic memory was tested by the Free and Cued Selective Reminding Test (FCSRT; Grober et al., 1988) which has be shown to predicts AD (Grober et al., 2000). Verbal fluency (Thurstone and Thurstone, 1964), executive function (Trail Making Test A and B; Tombaugh, 2004) and language production (Deloche and Hannequin, 1997) were evaluated. Depression was screened using the GDS (Yesavage, 1988). Disability was evaluated using Instrumental Activities of Daily Living (Lawton and Brody, 1969) and Activities of Daily Living (Katz et al., 1963) scales. Participants were then categorized as MCI according to international criteria (Petersen, 2004; Portet et al., 2006), amnestic MCI (aMCI) and non-aMCI (naMCI) subtypes (Petersen, 2004).

### MRI Protocol

The MRI protocol included 3D volumetric T1 weighted (W) an axial FLAIR T2W, an axial EG T2W and an axial T2W FSE. MRI analysis was centralized and analyzed by the Centre d’acquisition et de traitement d’images (CATI; Operto et al., 2016). Right and left hippocampal volume was obtained for each participant using automatic segmentation of the hippocampus (Chupin et al., 2009).The hippocampal volume was normalized using the following calculation: hippocampal volume/total brain volume × mean total brain volume.

### Statistical Analysis

General, medical and cognitive characteristics were first presented in the whole sample with percentages and numbers for categorical variables and means and standard deviations (SD) for continuous variables. Means and SD of variables of interest for the different values of each categorical variable were compared by analysis of variance. Pearson’s correlation coefficients and *p*-value were calculated for variables of interest and each continuous variable. Two multivariate linear regression models were performed with CSF SOM and NPY as dependent variables and biological variables significant associated with SOM and NPY in univariate analysis as independent variables. All Statistical analyses were performed using R Software version 3.1.1 (R Core Team (2014). R: a language and environment for statistical computing. R Foundation for Statistical Computing, Vienna, Austria^1^. In all analyses, the 2-sided α-level of 0.05 was used for significance testing.

## Results

Demographic, medical and cognitive characteristics of the 43 participants included are summarized in Table 1. The mean age (years (SD)) was 78.6 (5.4) with 53.5% of women. Fifty-six percent of the sample received at least a high school diploma, 20.9% had diabetes, 72.1% hypertension. The mean mini mental state examination (MMSE; SD) was: 27.2 (1.7)/30. MCI subtypes were aMCI and naMCI, in 69.8% and 30.2% of cases, respectively. APOE ε4 allele was present among 25% of the 43 tested participants. As shown in Table 1, these characteristics did not differ from the others participants suffering from MCI included in the BALTAZAR study at the Broca Hospital which were not tested for CSF SOM and NPY.

**Table 1:** General characteristics of the included MCI subjects compared to the non-included MCI in the Biomarker of AmyLoïd pepTide and AlZheimer’s disease Risk (BALTAZAR) study at the Broca hospital site.

General characteristics, mean (SD)	Whole sample	Non-included	Included	*p*1
	*N* = 325	*N* = 282	*N* = 43	
Age (years)	78.4 (5.7)	78.3 (5.8)	78.6 (5.4)	0.78
Female, % (*N*)	60.0 (195)	61.0 (172)	53.5 (23)	0.44
High school diploma, % (N)	56.7 (183)	56.8 (159)	55.8 (24)	0.99
Diagnosis, % (*N*)
Amnestic MCI	72.0 (234)	72.3 (204)	69.8 (30)	0.87
Non-amnestic MCI	28.0 (91)	27.7 (78)	30.2 (13)
Body mass index (kg^2^/m)	24.7 (3.8)	24.8 (3.9)	24.2 (3.5)	0.32
Hypertension, % (*N*)	70.1 (227)	69.8 (196)	72.1 (31)	0.89
Diabetes, % (*N*)	14.7 (47)	13.8 (38)	20.9 (9)	0.32
GDS/30	8.60 (5.38)	8.51 (5.35)	9.15 (5.57)	0.49
MMSE/30	26.4 (2.5)	26.3 (2.6)	27.2 (1.7)	0.04
Total cholesterol (mmol/l)	5.59 (1.17)	5.62 (1.15)	5.41 (1.26)	0.29
Triglycerides (mmol/l)	1.19 (0.53)	1.19 (0.55)	1.14 (0.43)	0.54
Albumin (g/l)	39.7 (2.7)	39.7 (2.7)	39.7 (2.6)	0.91
C-reactive protein (mg/l)	3.27 (6.03)	3.32 (6.30)	2.92 (3.85)	0.69
CSF SOM (fMol/mL)	-	-	543 (269)	-
CSF NPY (fMol/mL)	-	-	336 (173)	-
CSF Aβ_1–42_ (pg/ml)	899 (389)	885 (391)	948 (384)	0.37
CSF total -Tau (pg/ml)	424 (212)	416 (217)	455 (195)	0.30
CSF phospho-Tau (pg/ml)	66.9 (29.7)	66.3 (31.0)	69.0 (24.5)	0.62
IATI	1.42 (0.83)	1.43 (0.86)	1.37 (0.73)	0.70
Left hippocampus volume (cm^3^)	2.30 (0.56)	2.30 (0.58)	2.32 (0.47)	0.79
Right hippocampus volume (cm^3^)	2.37 (0.63)	2.36 (0.65)	2.45 (0.52)	0.42
Presence of APOE ε4-allele, % (*N*)	37.1 (118)	38.8 (108)	25.0 (10)	0.13

*MCI, Mild cognitive impairment; GDS, geriatric depression scale; MMSE, mini mental state examination; HDL, high density lipoprotein; LDL, low density lipoprotein; CSF, cerebrospinal fluid; SOM, somatostatin; NPY, neuropeptide Y; IATI, Innotest Amyloid Tau Index*.

The CSF SOM and NPY concentrations according to demographic, clinical, cognitive, biological (including APOE ε4 genotype) and imaging characteristics are summarized in Tables 2, 3, respectively. Factors associated with high CSF SOM concentrations were hypertension (*p* = 0.004), diabetes (*p* < 0.0001), low plasma total cholesterol (*p* = 0.004) and LDL cholesterol (*p* = 0.002), high CSF NPY concentrations (*p* = 0.03) and high CSF Aβ_1–42_ concentrations (*p* = 0.03; Table 2).

**Table 2:** CSF SOM according to demographic, clinical, cognitive, biological (including APOE ε4 genotype) and Magnetic Resonance Imaging (MRI) characteristics in MCI subjects.

Characteristics	*n*	CSF SOM (fmol/ml)	p*
		M (SD) or Pearson’s *r*	
Age		−0.0849	0.59
Gender			
Male	20	599 (323)	0.21
Female	23	495 (207)	
High school diploma		
No	19	502 (238)	0.38
Yes	24	576 (292)	
Diagnosis			
Amnestic MCI	30	540 (259)	0.92
Non-amnestic MCI	13	550 (303)	
Body mass index		0.168	0.28
Hypertension			
No	12	359 (226)	0.004
Yes	31	615 (253)	
Diabetes			
No	34	463 (213)	<0.0001
Yes	9	845 (251)	
GDS/30		−0.0334	0.84
MMSE/30		0.00251	0.99
Total cholesterol		−0.428	0.004
HDL cholesterol		−0.24	0.12
LDL cholesterol		−0.463	0.002
Triglycerides		0.232	0.13
Serum creatinine		0.139	0.37
Albumin		0.095	0.54
C-reactive protein		−0.148	0.34
CSF Neuropeptide Y		0.324	0.03
CSF Aβ_1–42_		0.341	0.03
CSF Total-Tau		0.0583	0.72
CSF phospho-Tau		0.0481	0.77
IATI		0.208	0.20
Left hippocampus volume (cm^3^)		−0.144	0.37
Right hippocampus volume (cm^3^)		−0.0626	0.70
Presence of APOE ε4-allele		
No APOE ε4-allele	30	585 (289)	0.07
1 or 2 APOE ε4-allele	10	399 (193)	

*ANOVA for categorical parameters or test based on Pearson’s product moment correlation coefficient (that follows a t-distribution with n-2 degrees of freedom) for continuous variables.*SOM, somatostatin; MCI, mild cognitive impairment; GDS, geriatric depression scale; MMSE, mini mental state examination; HDL, high density lipoprotein; LDL, low density lipoprotein; CSF, cerebrospinal fluid; IATI, Innotest Amyloid Tau Index*.

**Table 3:** CSF NPY according to demographic, clinical, cognitive, biological (including APOE ε4 genotype) and MRI characteristics in MCI subjects.

Characteristics	*n*	CSF NPY (fmol/ml)	p*
		M (SD) or Pearson’s *r*	
Age		−0.155	0.32
Gender			
Male	20	393 (174)	0.04
Female	23	287 (160)	
High school diploma		
No	19	303 (173)	0.26
Yes	24	363 (172)	
Diagnosis			
Amnestic MCI	30	356 (169)	0.25
Non-amnestic MCI	13	290 (179)	
Body mass index		0.184	0.24
Hypertension			
No	12	290 (196)	0.28
Yes	31	354 (163)	
Diabetes			
No	34	303 (170)	0.01
Yes	9	463 (122)	
GDS/30		−0.168	0.30
MMSE/30		−0.138	0.38
Total cholesterol		−0.42	0.005
HDL cholesterol		−0.441	0.003
LDL cholesterol		−0.344	0.02
Triglycerides		0.23	0.14
Serum creatinine		0.373	0.01
Albumin		0.194	0.21
C-reactive protein		−0.221	0.15
CSF SOM		0.324	0.03
CSF Aβ_1–42_		−0.0714	0.66
CSF total -Tau		0.366	0.02
CSF phospho-Tau		0.399	0.01
IATI		−0.167	0.30
Left hippocampus volume (cm^3^)		0.0405	0.80
Right hippocampus volume (cm^3^)		0.0763	0.64
Presence of APOE ε4-allele		
No APOE ε4-allele	30	343 (178)	0.46
1 or 2 APOE ε4-allele	10	295 (178)	

*ANOVA for categorical parameters or test based on Pearson’s product moment correlation coefficient (that follows a t-distribution with n-2 degrees of freedom) for continuous variables. *NPY, neuropeptide Y; MCI, mild cognitive impairment; GDS, geriatric depression scale; MMSE, mini mental state examination; HDL, high density lipoprotein; LDL, low density lipoprotein; CSF, cerebrospinal fluid; IATI, Innotest Amyloid Tau Index*.

Factors associated with high CSF NPY concentrations were male gender (*p* = 0.04), diabetes (*p* = 0.01), low plasma total cholesterol (*p* = 0.005), HDL cholesterol (*p* = 0.003) and LDL cholesterol (*p* = 0.02), high serum creatinine (*p* = 0.01); high CSF T-Tau (*p* = 0.02) and P-Tau concentrations (*p* = 0.01) and high CSF SOM concentrations (*p* = 0.03; Table 3).

**Figure 1:**
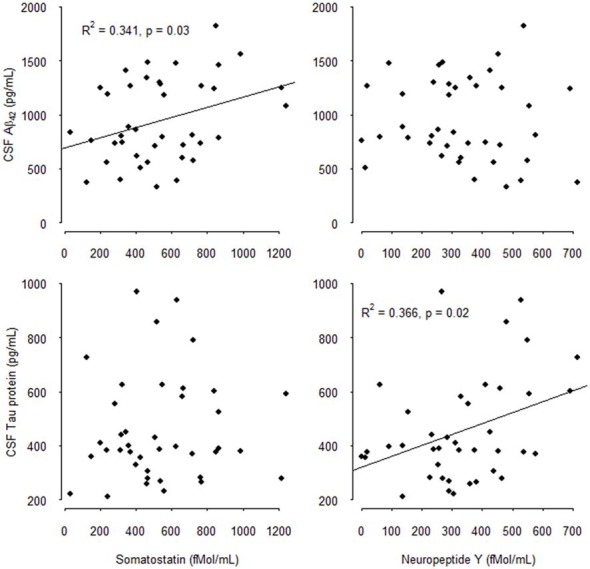
Correlations between cerebrospinal fluid (CSF) Somatostatin (SOM), Neuropeptide Y (NPY), Amyloid β_1–42_ (Aβ_1–42_), and total-Tau (T-Tau) in mild cognitively impairment (MCI) subjects.

The results of the multivariate analysis are presented in Tables 4A,B including significant biological variables in univariate analysis. CSF SOM concentrations remained independently associated with CSF Aβ_1–42_ concentrations (*p* = 0.01) and blood total cholesterol concentrations (*p* = 0.03) and CSF NPY remained significantly associated with CSF T-Tau (*p* = 0.03). CSF SOM and CSF NPY were no more significantly associated. The Figure 1 illustrates the correlations between CSF SOM, NPY, Aβ_1–42_ and T-Tau in included subjects.

**Table 4A:** Factors associated with CSF SOM.

Factors	β (SE)	*t* value	
CSF NPY	0.394 (0.236)	1.672	0.10
Total cholesterol	−72.0 (32.3)	−2.229	0.03
log CSF total-Tau	231 (86)	2.681	0.01

**Table 4B:** Factors associated with CSF NPY.

Factors	β (SE)	*t* value	
CSF SOM	0.114 (0.099)	1.146	0.26
Sex	−31.9 (24.6)	−1.298	0.20
Total cholesterol	−72.0 (32.3)	−2.229	0.03
log CSF total-Tau	140 (63)	2.217	0.03

## Discussion

The present results indicate that high CSF SOM and high CSF Aβ_1–42_ concentrations on the one hand and high CSF NPY and high CSF Tau and P-Tau concentrations on the other hand, are correlated in a small population of elderly subjects suffering from MCI. Moreover, high CSF SOM concentrations are also related to diabetes and hypertension and low plasma cholesterol level. High CSF NPY concentrations are related to male gender, diabetes, and low plasma cholesterol.

Tau and P-Tau are released in the CSF very early in the AD process when neuronal damage occurs (Sperling et al., 2011). The correlation between CSF NPY and CSF T-Tau and P-Tau may reflect an early loss of a subset of hippocampal NPY/SOM neurons containing neurofibrillary tangles (Chan-Palay, 1987). However, the majority of cortical NPY-containing neurons are spared in AD (Gaspar et al., 1989; Unger and Lange, 1992). NPY may also stimulate autophagy of neurons containing neurofibrillary tangles to allow T-Tau and P-Tau clearance (Duarte-Neves et al., 2016). NPY is contained in GABA neurons (Aoki and Pickel, 1989) mainly in the amygdala, the hippocampus and the striatum (Duarte-Neves et al., 2016). NPY is neurotrophic, regulates calcium homeostasis, stimulates autophagy and attenuates inflammation processes involved in AD pathology (Duarte-Neves et al., 2016). In the APP23 mouse model, NPY is increased in mice cortex and hippocampus (Diez et al., 2000, 2003) which can reflect NPY up regulation (Duarte-Neves et al., 2016). In another AD transgenic mouse model, presenting both Presenilin 1 and APP mutations (PS1 × APP), figuring a later AD stage, hippocampal NPY neuronal densities in hippocampus are decreased (Ramos et al., 2006). Taken together, it may be suggested that, in earlier states, NPY levels are increased, and then disease progression results in NPY-interneurons loss leading to the decrease in NPY levels (Duarte-Neves et al., 2016). In AD brains, morphology of NPY-positive neurons were initially reported as altered in the cerebral cortex and in the hippocampus (Chan-Palay, 1987) and the density of NPY binding sites is decreased in those regions (Martel et al., 1990). Nevertheless, studies about NPY levels in AD CSF provided controversial results: some of them report that CSF NPY is decreased in AD compared than controls (Martignoni et al., 1992; Edvinsson et al., 1993) whereas this was not replicated in others (Atack et al., 1988; Heilig et al., 1995).

In contrast to NPY, the SOM neuronal systems of the hippocampus and cortical areas appear to be especially sensitive to aging (Martel et al., 2012; Stanley et al., 2012; French et al., 2017; Rozycka and Liguz-Lecznar, 2017; ) and cortex and hippocampal SOM concentrations are further decreased in brains of demented patients (Epelbaum et al., 1983). In frontal and temporal cortical areas, a marked decrease in the density of SOM containing neurons is observed in AD, whereas density of NPY-interneurons is unchanged (Gaspar et al., 1989). Accordingly, the proportion of single-labeled SOM neurons decreases and correlates with senile plaques, indicating that single SOM-interneurons, in cortical layers II-III and V, are preferentially affected relative to co-localized SOM-NPY neurons (Gaspar et al., 1989). More recent neuro-pathological studies show that SOM-interneurons, in the anterior olfactory nucleus, the piriform cortex (with co-localization of Aβ_1–42_; Saiz-Sanchez et al., 2015), the amygdala and entorhinal cortex are early involved in AD process (Saiz-Sanchez et al., 2016). The association between CSF Aβ_1–42_ and SOM concentrations may be explained by the fact that Aβ seems to specially affect SOM neurons. SOM and Aβ_1–42_ have complex physical interactions (Wang et al., 2017). Moreover SOM increases neprilysin activity involved in Aβ degradation (Saito et al., 2005; Epelbaum et al., 2009). SOM also regulates Aβ_1–42_ catabolism by modulating insulin degrading enzyme proteolytic activity (Tundo et al., 2012). Furthermore, Aβ intra-hippocampal injections in rats induced aberrant inhibitory network activity associated with an impairment of hippocampal memory processes (Villette et al., 2010). This effect can be explained by the selective disappearance of hippocampal projecting neurons containing SOM (Villette et al., 2012).

The relationships between CSF neuropeptide concentrations, hypertension, diabetes and fasting blood cholesterol levels are particularly interesting in view of the metabolic and non-clinical abnormalities, such as alterations in body weight and neuroendocrine functions, which often precede the cognitive decline (Low and Singer, 2008; McGrath et al., 2017). A polymorphism in the NPY gene resulting in a change of leucine 7 to proline in the signal peptide is associated with elevated cholesterol levels, higher alcohol consumption, and may be a risk factor for various metabolic and cardiovascular diseases (de Luis et al., 2016). Another one, the IVSI-100G/T polymorphism, influences cholesterol levels in the CSF and this effect is more pronounced in a German population of AD patients than in their controls (Kolsch et al., 2006). However, it does not influence plasma cholesterol levels. In the case of SOM, fasting plasma concentration of N-Terminal proSOM, a surrogate marker of SOM —given the very short half-life of the bioactive peptide— independently predicts development of coronary artery disease and both all-cause and cardiovascular mortality in the 5,389 fasting participants of the population-based study Malmö Preventive Project (Hedback et al., 2016) and in the 8,134 participants from the Prevention of Renal and Vascular End-stage Disease (PREVEND) study in Groningen (Abbasi et al., 2017). Moreover, higher levels of circulating N-terminal-prosomatostatin are associated with increased incidence of vascular dementia in the prospective population-based Malmö Preventive Project (Holm et al., 2017). The association between high CSF SOM concentrations and low plasma cholesterol levels in MCI subjects may be associated with weight loss and lower cholesterol level as observed in the early stages of AD (Duron and Hanon, 2008). Nevertheless, CSF SOM concentrations do not necessarily reflect plasma SOM concentrations.

SOM and its receptors are potential pharmacological targets for AD. FK962, which promotes SOM production, in the brain, co-administered with donepezil, enhances cognition in rats (McCarthy et al., 2011). In AD patients (Craft et al., 1999) as in cognitively normal subjects (Watson et al., 2009), intravenous administration of octreotide, a SOM 1 receptor agonist, facilitates memory.

The present study is limited by its monocentric design, the small sample size and neuropsychological diagnosis heterogeneity. Moreover, in the subsample studied, mean MMSE was slightly higher than in non included subjects’ mean MMSE. It should be noted that MMSE provides only a global assessment of cognition, not precise enough to detect slight differences, At any rate, MMSE was neither correlated with CSF SOM nor CSF NPY. Nevertheless, it would be interesting to know whether the concentrations of both peptides in MCI cases are altered as compared to a control population but this raises a number of ethical issues.

Nevertheless, this is the first report of a relationship between CSF SOM and NPY, two peptides produced by subsets of GABergic interneurons, and the CSF AD markers (CSF Aβ_1–42_ and T-Tau) in an exhaustively phenotyped population.

We report for the first time an independent relationship between CSF SOM and NPY, two peptides known to be affected in AD pathophysiology, and CSF markers of the two pathophysiological processes in AD: Aβ_1–42_ deposition and hyper phosphorylated Tau aggregation. The present study needs to be replicated in a larger sample size. If those results are confirmed additional interest of CSF SOM and NPY to already known AD biomarkers could be tested.

## Author Contributions

ED, DG, AG, CB, BA, and SL performed measurements and collected data. J-SV performed statistical analysis. FP, LB, SS-M, A-SR, OH, and JE supervised the study. SB collected data revised the manuscript.

## Conflict of Interest Statement

The authors declare that the research was conducted in the absence of any commercial or financial relationships that could be construed as a potential conflict of interest.

## Footnotes

^1^http://www.R-project.org/
