# Corrigendum: Somatostatin and Neuropeptide Y in Cerebrospinal Fluid: Correlations With Amyloid Peptides Aß_1−42_, and Tau Proteins in Elderly Patients With Mild Cognitive Impairment

**DOI:** 10.3389/fnagi.2019.00011

**Published:** 2019-02-19

**Authors:** Emmanuelle Duron, Jean-Sébastien Vidal, Dominique Grousselle, Audrey Gabelle, Sylvain Lehmann, Florence Pasquier, Stéphanie Bombois, Luc Buée, Bernadette Allinquant, Susanna Schraen-Maschke, Christiane Baret, Anne-Sophie Rigaud, Olivier Hanon, Jacques Epelbaum

**Affiliations:** ^1^AP-HP, Hôpital Broca, Service de Gériatrie, Paris, France; ^2^Université Sorbonne Paris Cité, UMR-S894, INSERM Université Paris Descartes, Centre de Psychiatrie et Neuroscience, Paris, France; ^3^APHP, Hôpital Paul Brousse, Service de Gériatrie du Dr Karoubi, Villejuif, France; ^4^Université Paris-Sud 11, Centre de Recherche en Épidemiologie et Santé des Population-Depression et Antidépresseurs, INSERM UMR-1178, Le Kremlin-Bicêtre, France; ^5^Université Paris Descartes, Sorbonne Paris Cité, Paris, France; ^6^Memory Research and Resources Center, Gui de Chauliac Hospital, University of Montpellier, Montpellier, France; ^7^Laboratoire de Biochimie Protéomique Clinique, CHU Montpellier, University of Montpellier, INSERM, Montpellier, France; ^8^University of Lille, INSERM 1171, CHU, Centre Mémoire (CMRR) Distalz, Lille, France; ^9^Univ Lille, Inserm, UMRS 1172, LabEx DISTALZ, Lille, France; ^10^UF de Neurobiologie, Centre Biologie Pathologie du CHU-Lille, Lille, France; ^11^MECADEV UMR 7179 CNRS, Muséum National d'Histoire Naturelle, Paris, France

**Keywords:** somatostatin, neuropeptide Y, peptides Aß_1−42_, tau proteins, cerebrospinal fluid, mild cognitive impairment

In the published article, there was an error in affiliation 9. Instead of “UF de Neurobiologie, Centre Biologie Pathologie du CHU-Lille, Lille, France,” it should be “Univ Lille, Inserm, UMRS 1172, LabEx DISTALZ, Lille, France.”

Additionally, Stéphanie Bombois was not included as an author in the published article. The corrected Author Contributions Statement appears below.

“ED, DG, AG, CB, BA, and SL performed measurements and collected data. J-SV performed statistical analysis. FP, LB, SS-M, A-SR, OH, and JE supervised the study. SB collected data revised the manuscript; Final approval of the version to be published; and is an Agreement to be accountable for all aspects of the work in ensuring that questions related to the accuracy or integrity of any part of the work are appropriately investigated and resolved.”

The authors apologize for these errors and state that this does not change the scientific conclusions of the article in any way. The original article has been updated.

## Conflict of Interest Statement

The authors declare that the research was conducted in the absence of any commercial or financial relationships that could be construed as a potential conflict of interest.

